# Risk for *Pneumocystis carinii* Transmission among Patients with Pneumonia: a Molecular Epidemiology Study

**DOI:** 10.3201/eid0901.020141

**Published:** 2003-01

**Authors:** Elodie Senggen Manoloff, Patrick Francioli, Patrick Taffé, Guy van Melle, Jacques Bille, Philippe M. Hauser

**Affiliations:** * Centre Hospitalier Universitaire Vaudois, Lausanne, Switzerland; Institut Universitaire de Médecine Sociale et Préventive, Lausanne, Switzerland

**Keywords:** *Pneumocystis carinii*, pneumonia, molecular epidemiology, typing, disease transmission, dispatch

## Abstract

We report a molecular typing and epidemiologic analysis of *Pneumocystis carinii* pneumonia (PCP) cases diagnosed in our geographic area from 1990 to 2000. Our analysis suggests that transmission from patients with active PCP to susceptible persons caused only a few, if any, PCP cases in our setting.

*Pneumocystis carinii* pneumonia (PCP) is an important cause of illness and death in immunocompromised patients, specifically HIV-infected patients, transplant recipients, and patients with oncohematologic diseases. In the absence of a reliable method of in vitro culture of the pathogen, many aspects of PCP epidemiology remain to be elucidated (*1*). Because of the evidence that contact with *P. carinii* occurs early in life, clinical infection in adults was thought to be mostly the result of reactivation of latent organisms. However, this concept has been challenged by evidence of the occurrence of de novo infection in HIV-infected persons (*2*,*3*). Possible sources include the environment, asymptomatic carriers, unrecognized infections, and patients with active PCP. Carriage by some persons with severe immunosuppression (*4*) or with chronic pulmonary disease (*5*) has been described recently. Direct transmission of *P. carinii* from patients with active PCP to susceptible persons is suggested by numerous reports of clusters of PCP cases, as well as by the demonstration of transmission of *P. carinii* in animal models (*6*). However, recent molecular typing failed to support this hypothesis in some studies (*7*–*9*). Indeed, different molecular *P. carinii* types were found in members of three different couples diagnosed with PCP (*7*), and the types were often different within clusters of PCP cases (*8*,*9*). Using molecular typing and epidemiologic analysis, we investigated the possibility of transmission of *P. carinii* between PCP patients who were seen in our region (about 500,000 inhabitants) during recent years. With few exceptions, all PCP patients are diagnosed at or referred to our hospital, Centre Hospitalier Universitaire Vaudois.

From October 1990 to August 2000, 1,299 bronchoalveolar lavage specimens were examined at our hospital by using the Gomori staining method; 230 (18%) were positive for *P. carinii* (including eight recurrent episodes), among which 131 (57%) were available for typing (127 patients, four recurrent episodes). The typing system consisted of amplification by polymerase chain reaction (PCR) of four variable regions of the *P. carinii* genome, followed by the detection of the polymorphisms using the single-strand conformation polymorphism technique (SSCP). The four genomic regions are: the internal transcribed spacer number 1 of the nuclear rRNA genes operon, the intron of the nuclear 26S rRNA gene, the variable region of the mitochondrial 26S rRNA gene, and the region surrounding the intron number 6 of the beta-tubulin gene. These four genomic regions were shown to be stable over prolonged periods of time by using SSCP (*10*). The interpretation of SSCP results for typing has been described (*11*,*12*). A *P. carinii* type is defined by a combination of four alleles corresponding to the four genomic regions. This system has been validated (*10*–*12*). The SSCP typing system is easier and faster than DNA sequencing of multiple loci and allows the analysis of large collections of specimens. Moreover, this system detects a higher proportion of coinfections than typing by DNA sequencing of several genetic loci, probably because it is more sensitive at detecting alleles present in low amounts (*11*,*13*). This higher rate of detection is important for epidemiologic studies. The disadvantage of SSCP is that specimens containing more than two *P. carinii* types (about 30% of the specimens) cannot be typed because of the complexity of the alleles’ configuration (*11*).

The ages of the 127 patients ranged from 25 to 82 years (median 38), and 93 (73%) were men. Seventy-three percent (93/127) of the patients were HIV infected, and 27% were immunosuppressed from other conditions. Twenty-three (18%) of the 131 PCP episodes corresponded to an infection with a single *P. carinii* type, 66 (50%) to a coinfection with two types, and 42 (32%) to a coinfection with more than two types. The four patients with two PCP episodes were infected with different types at each episode. Altogether, 35 different PCR-SSCP types were observed (numbered as in a previous publication [*10*]).

To evaluate the proportion of PCP cases that could have resulted from transmission of *P. carinii* from a patient with active PCP to a susceptible person, we analyzed the distribution over time of the *P. carinii* types observed in the patients carrying one or two types (89 episodes, 39% of the cases in the period). Described clusters of PCPs (*14*–*16*) suggest that the incubation period of *P. carinii* infection may range from 3 to 12 weeks, which is also in accordance with experiments in animals (*17*,*18*). Accordingly, we hypothesized that the incubation period of a newly acquired infection would range from 3 weeks to 3 months and that a patient with PCP might be infectious from 1 month before diagnosis until the end of treatment (usually 3 weeks after diagnosis). A patient can be both a receptor of *P. carinii* and a donor. The Figure represents the receptor-donor period for each *P. carinii* type identified in a patient (a patient with a coinfection has two types). The distribution of the types over time was relatively homogeneous, suggesting the absence of outbreaks due to a single type. In only 19 instances did the periods of two or more isolates of a given type overlap.

**Figure:**
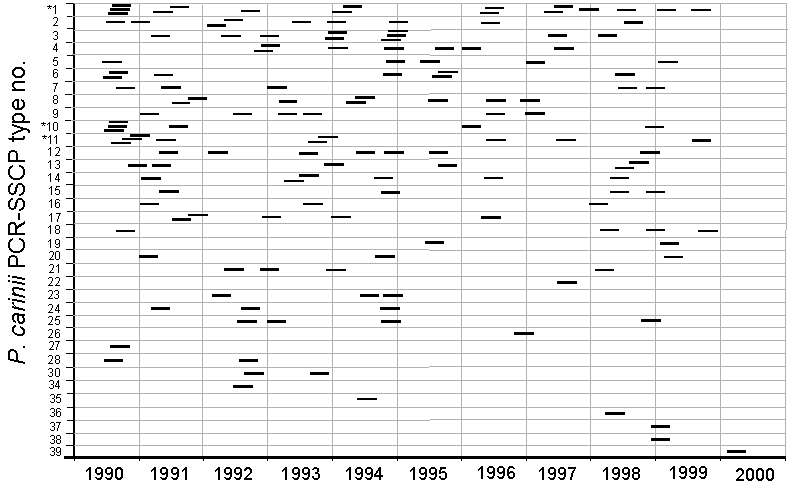
Distribution over time of *Pneumocystis carinii* types, as determined by polymerase chain reaction (PCR) – single-strand conformation polymorphism technique (SSCP), observed in Centre Hospitalier Universitaire Vaudois. Each occurrence is represented as a bar corresponding to the period of 15 weeks from 3 months before to 3 weeks after PCP diagnosis. Starred types may exhibit excessive clustering (see text).

To investigate whether these overlapping periods could reflect transmission of *P. carinii* between the members of the cluster, we calculated, for each type, the multinomial probability that the observed clusters occurred by chance alone using Monte-Carlo simulation experiments (we assumed uniform distribution over time). For types 1, 10, and 11, the probability was small (4.6%, 5.6%, and 5.4%, respectively). This probability is even smaller when we consider only 1990–1995 (before antiretroviral tritherapy was introduced), a period during which more cases were observed (1.5%, 2.3%, and 1.5%, respectively). This probability suggests that some clusters may indeed be the result of interhuman transmission of *P. carinii* (or of infection from a common source). However, 16 of the 19 clusters involved patients infected with different coinfecting types, implying that interhuman transmission was less likely (either the donor would have not transmitted both types or the receptor would have acquired one type elsewhere, or both).

We further investigated the possibility of transmission between members of each cluster using available epidemiologic data limited to the location of patients’ residence and documented time in the hospital. None of the patients involved in the 19 clusters lived in the same city sector or the same town. To investigate the possibility of encounters in the hospital, timing and location of consultations and hospitalizations were determined by review of the patients’ medical charts and the outpatient clinic’s schedule. An encounter was considered compatible with an interhuman transmission of *P. carinii* if an “infectious” and a “susceptible” patient (as defined above) were present in the same ward or facility of the hospital on the same day. Analyses of all identified clusters did not reveal any hospital encounters. Because the period at risk of acquisition and transmission chosen might have been too restrictive, we also analyzed four clusters of patients with identical typing results but with PCP episodes occurring up to 10 months apart. Again, no encounter in the hospital, as defined above, was found.

Because the analyzed specimens represent only 39% (89/230) of the PCP episodes of the period, the possibility of a selection bias can be raised. Such bias cannot be firmly excluded but statistical analysis argues against it, at least for the period before antiretroviral tritherapy was introduced (a period covering 51% [87/171] of the specimens available). Statistical analysis showed that the distribution over time of the available specimens was not significantly different from that of the cases that were unavailable (Wilcoxon rank sum test; p=0.55), suggesting that the results for this period are representative of the overall situation. On the other hand, the two distributions for the period from 1996 to 2000 were different because the proportion of specimens stored for typing increased regularly during the period (p=0.07; 75% [44/59] of the specimens available).

Our study reports on a large number of PCP episodes observed for >10 years. Although our retrospective analysis does not allow us to exclude encounters of the clustered cases outside the hospital, the results strongly suggest that transmission of *P. carinii* by a patient with active PCP to a susceptible person contributed to only a very small number, if any, of the PCP cases in our geographic area during this period. The broad diversity of observed types suggests that the patients acquired *P. carinii* from multiple unknown sources not addressed in the present study. Moreover, hospital epidemiologic data and molecular typing did not provide evidence of transmission of *P. carinii* inside the hospital between members of clusters infected with the same *P. carinii* type. Thus, the main source of *P. carinii* is unlikely to be represented by patients with active PCP.
